# Differential risks of syringe service program participants in Central Ohio: a latent class analysis

**DOI:** 10.1186/s12954-023-00824-8

**Published:** 2023-07-28

**Authors:** Kyle J. Moon, Ian Bryant, Anne Trinh, Kathryn A. Hasenstab, Brittany Carter, Rick Barclay, Saira Nawaz

**Affiliations:** 1 Center for Health Outcomes and Policy Evaluation Studies (HOPES), The Ohio State University College of Public Health, 381 Cunz Hall, 1841 Neil Avenue, Columbus, OH 43210 USA; 2 Equitas Health, Columbus, OH USA; 3 Division of Health Services Management and Policy, The Ohio State University College of Public Health, Columbus, OH USA

**Keywords:** LCA, Harm reduction, Needle and syringe exchange, Overdose, HIV, Hepatitis C, Drug use, Substance misuse, Opioids, Stimulants, SSP

## Abstract

**Background:**

Significant heterogeneity exists among people who use drugs (PWUD). We identify distinct profiles of syringe service program (SSP) clients to (a) evaluate differential risk factors across subgroups and (b) inform harm reduction programming.

**Methods:**

Latent class analysis (LCA) was applied to identify subgroups of participants (*N* = 3418) in a SSP in Columbus, Ohio, from 2019 to 2021. Demographics (age, sex, race/ethnicity, sexual orientation, housing status) and drug use characteristics (substance[s] used, syringe gauge, needle length, using alone, mixing drugs, sharing supplies, reducing use, self-reported perceptions on the impact of use, and treatment/support resources) were used as indicators to define latent classes. A five-class LCA model was developed, and logistic regression was then employed to compare risk factors at program initiation and at follow-up visits between latent classes.

**Results:**

Five latent classes were identified: (1) heterosexual males using opioids/stimulants with housing instability and limited resources for treatment/support (16.1%), (2) heterosexual individuals using opioids with stable housing and resources for treatment/support (33.1%), (3) individuals using methamphetamine (12.4%), (4) young white individuals using opioids/methamphetamine (20.5%), and (5) females using opioids/cocaine (17.9%). Class 2 served as the reference group for logistic regression models, and at the time of entry, class 1 was more likely to report history of substance use treatment, overdose, HCV, sharing supplies, and mixing drugs, with persistently higher odds of sharing supplies and mixing drugs at follow-up. Class 3 was more likely to report history of overdose, sharing supplies, and mixing drugs, but outcomes at follow-up were comparable. Class 4 was the least likely to report history of overdose, HCV, and mixing drugs, but the most likely to report HIV. Class 5 was more likely to report history of substance use treatment, overdose, HCV, sharing supplies, and mixing drugs at entry, and higher reports of accessing substance use treatment and testing positive for HCV persisted at follow-up.

**Conclusions:**

Considerable heterogeneity exists among PWUD, leading to differential risk factors that may persist throughout engagement in harm reduction services. LCA can identify distinct profiles of PWUD accessing services to tailor interventions that address risks, improve outcomes, and mitigate disparities.

**Supplementary Information:**

The online version contains supplementary material available at 10.1186/s12954-023-00824-8.

## Background

The drug overdose epidemic is one of the foremost public health crises in the USA, reaching devastating heights over the course of the COVID-19 pandemic [1, 2]. Data indicate that 2021 was the deadliest year on record, with an estimated 107,622 lives lost to overdose [3], presenting a noteworthy economic burden [4].

Harm reduction is key to addressing and responding to the worsening overdose crisis [5, 6]. A pragmatic philosophy, harm reduction (a) recognizes drug use is inevitable in any society, (b) acknowledges the dignity of people who use drugs (PWUD), and (c) aims to reduce the negative social and health consequences associated with drug use [5, 7, 8]. The 1980s brought about the advent of harm reduction in the USA, during which PWUD organized the implementation of syringe service programs (SSPs). In the face of the emergent AIDS epidemic that disproportionately impacted PWUD [5, 9], SSPs were instituted to prevent the transmission of human immunodeficiency virus (HIV) as well as hepatitis C virus (HCV). Evidence has mounted in the decades since that demonstrates the effectiveness of SSPs in reducing transmission of infectious diseases [10]; facilitating linkages-to-care for substance use disorder (SUD) treatment, counseling, and primary care [11]; and preventing overdose deaths [12], all of which contributes to the well-established cost-effectiveness of SSPs [13]. SSPs today have evolved into multi-service organizations [9, 14], offering a wide range of education and health and social services, including but not limited to overdose education, counseling and treatment referrals, drug checking, primary care linkage, case management, and infectious diseases and sexually transmitted infection (STI) testing [14, 15]. The range of services provided by SSPs are key to their effectiveness, as PWUD are not a monolith [16, 17].

Distinct subgroups of PWUD, based on patterns of drug use and socio-demographic characteristics [18–20], have been identified in studies employing latent class analysis (LCA). LCA is a statistical modeling technique that identifies homogenous subgroups within a heterogeneous population, which can distinguish typologies of individuals that share characteristics and exposures to hazards [21, 22]. Beyond mere identification of subgroups of PWUD, LCA has been used to evaluate associations between profiles of PWUD and risk of both HIV acquisition [19] and overdose [23]. Previous studies have largely been limited to the emergency room [20] or community corrections programs [19], and those using community samples have noted varying levels of (dis)engagement in harm reduction programs across PWUD [23–26]. SSPs typically serve client populations that are at high risk of overdose [6], exacerbated by the introduction of fentanyl and xylazine [27], but are unlikely to access healthcare services due to stigma [6, 28]. Despite similarities in structural barriers, client populations are largely heterogeneous [29–31], posing a challenge to harm reduction organizations in tailoring programs and developing targeted interventions to best serve clients and meet their unique needs [16, 31].

Extant literature has articulated the ways that social locations–including gender [32], race [33], sexual orientation [34], age [35], and housing status [36]–shape the risk environment of PWUD and ultimately influence health and drug outcomes [37]. The aim of the present study, therefore, is to apply LCA to (1) identify distinct profiles of PWUD participating in a SSP in Central Ohio, based on demographic and drug use characteristics, and (2) evaluate associations between latent class membership and risk factors (substance use treatment, overdose, HIV, HCV, mixing drugs, sharing injection equipment, and using alone) at the time of SSP intake and follow-up visits. Previous studies have applied LCA methodology to identify and describe heterogeneity among PWUD [31, 38–40]. We build upon this work by evaluating infectious disease and drug-related risks in tandem and using longitudinal data to assess outcomes, with the goal of informing future harm reduction programming.

## Methods

### Study design and setting

Safe Point (Columbus, Ohio) is a SSP supported by Columbus Public Health and operated by Equitas Health, a community health center serving those affected by or at risk for HIV. Safe Point is the only SSP in Central Ohio, serving the metropolitan Franklin County and the surrounding suburban and partially rural counties. Programmatic data show that the majority of clients are non-Hispanic/Latinx white, heterosexual men between 25 and 44 years of age (Additional file 1: Table 1A). Harm reduction services offered by Safe Point include: (a) syringe exchange, in which new syringes are dispensed in exchange for safe disposal of used syringes; (b) overdose prevention education; (c) testing for HIV, HCV, and other STIs; (d) provision of naloxone, fentanyl test strips, supplies for safer injection and blood-borne illness prevention (e.g., cookers, cottons, tourniquets, sharps containers), and safer sex supplies (e.g., condoms, lubricant); (e) health navigation and case management (e.g., insurance enrollment, public benefits assistance, housing assistance); and (f) referrals and linkages to care for primary care, substance use treatment, dental care, and social services.

Interviews are only conducted at the participant’s first visit and every three months thereafter, unless requested otherwise. Full interviews were discontinued in spring 2020–and gradually reintroduced at the beginning of 2021–due to amended COVID-19 protocols. Safe Point participants select syringes (large [27 gauge] or small [28–31 gauge]) as well as any other supplies for safer injection and/or safer sex, including tourniquets, cottons, cookers, sharps containers, fentanyl test strips, naloxone, alcohol swabs, ointment, bandages, condoms, and lubricant. Quick exchange services are available for participants’ interim visits (i.e., when an interview is not required because one has been conducted within 3 months).

Programmatic data collected by Safe Point between 2019 and 2021 were made accessible to Ohio State University (OSU) researchers for this study. Safe Point operates as an anonymous program, in that no identifying information is collected from participants. At the time of program enrollment, participants receive a unique ID that is used for all visits, enabling assessment of participant outcomes over time. All data shared with and analyzed by OSU researchers were de-identified. As such, this study was determined to be exempt human subjects research by the Institutional Review Board (IRB) at the Ohio State University (IRB # 2023E0055).

### Data collection

Individuals that participate in Safe Point enter the program by completing an intake interview with a staff member or trained volunteer, during which they assess the participant’s demographics, drug use characteristics, risk factors, and personal goals or motivations for participating in Safe Point programming. Data are all self-reported, with the exception of syringe size and needle length, which is recorded by staff or trained volunteers based on what is distributed. Interview questions are asked orally by staff or experienced volunteers and recorded on paper; data entry is performed post hoc by staff members, who enter data into a secure, electronic database. In April 2021, all data entry was performed directly into the secure, electronic database.

Demographic data include the following indicators: age (years), gender identity (male, female, transgender, non-binary), race (American Indian or Alaska Native, Asian, Black or African American, Native Hawaiian or Other Pacific Islander, White), ethnicity (Hispanic/Latinx or non-Hispanic/Latinx), sexual orientation (Heterosexual or Lesbian, Gay, Bisexual, Transgender, Queer [LGBTQ]), and current housing status (“stable” or “homeless”).

Data on drug use characteristics are used to assess participant risk and identify what additional services (e.g., housing assistance, medical referral) and/or education (e.g., alternating sites of injection) might be necessary. Data include the following: substances presently used (cocaine, opioids, methamphetamine), syringe size (large [27 gauge] or small [28–31 gauge]), and needle length (long [1/2″] or short [5/16″]).

Risk factor data include the following: treatment history (whether or not they have been in any substance use treatment program or support group in the past year), overdose history (whether or not they have overdosed in the past year), HCV test result (positive, negative, or unknown), HIV test result (positive, negative, or unknown), whether they share injection equipment (yes/no), whether they mix drugs (yes/no), whether they typically use alone (yes/no), whether they perceive using to interfere with their job or personal life (yes/no), whether they are trying to cut down on how much they use (yes/no), and whether they have trusted resources for treatment and/or support if they want to stop using (yes/no). These indicators are used at intake and at follow-up visits requiring an interview (≥ 3 months since initiating services).

### Data and statistical analysis

Participant ID was used to identify number of unique participants over the time period and to assess number of visits at the individual level. An exploratory LCA was performed to identify distinctive subgroups of participants within a heterogeneous sample of PWUD accessing services at Safe Point. LCA is a method of statistical modeling that uses independently observed categorical data to identify distinct yet homogeneous subgroups within heterogeneous populations [21, 41]. Conceptualizations of the risk environment and structural vulnerability guided the selection of indicator variables used to define the latent classes [33–37, 42, 43]. Sociodemographic characteristics (sex, race/ethnicity, age, sexual orientation, and housing status) were selected, recognizing (a) how race, gender identity, sexual orientation, age, and housing status shape the risk environment [33–37, 44–46], (b) disproportionate overdose mortality rates among racial and ethnic minorities and homeless adults [47, 48], and (c) disparities in treatment access for substance use and infectious diseases [49, 50]. Preferences related to injection equipment were also included because (a) larger syringes are often used by individuals with longer histories of substance use because of scar tissue and overall vein health [51] and (b) shorter needles can be used for skin popping and injecting into the hands, feet, and superficial veins, all of which can increase the risk of abscesses and skin and soft tissue infections [51, 52]. Similarly, characteristics of substance use were included because the types of substances used, whether drugs are used in combination, frequency of use, perceived support, and injection behaviors (using alone, sharing injection equipment) all influence risk. Model indicators were shared with leadership at Safe Point to assess the relevance and rationale for including each variable. After selecting indicator variables, five models were developed, each with a different number of latent classes. The final model was selected after evaluating model fit using the Bayesian information criterion (BIC), where lower values indicate better fit [22, 42].

Using the model with the best fit, the probability of membership is estimated for each class for each individual, with an individual’s membership ultimately being assigned to the class with the highest probability measure. Logistic regression models were developed to evaluate the association of latent class membership with risk factors at (a) the time of entry and (b) follow-up. Safe Point requires an interview after 3 months have elapsed since intake, but a myriad of factors influences participation frequency. Some follow-up visits were conducted 3 months after intake, while others were conducted after 6–12 months. Some clients had multiple follow-up visits during the study period, and in this case, data were aggregated into a composite follow-up visit to assess change from the intake visit to follow-up. Statistical significance of regression model predictors was assessed with the Wald test. All statistical analyses were performed with R Statistical Software, version 3.6.2 (R Foundation for Statistical Computing).

## Results

### Sociodemographic characteristics

The dataset provided by Safe Point contained 7890 unique individuals and 28,337 visits. LCA data were limited to individuals’ first interview between 2019 and 2021 (*n* = 5084) with data complete for all LCA indicator variables (*n* = 3418). Data for logistic regression models were limited to those initiating services at Safe Point between 2019 and 2021 (i.e., new clients), netting 3020 unique individuals for whom we evaluated associations between latent class membership and risk factors at the time of program enrollment. Of these individuals, 377 (13%) had > 1 visit and were included in a second regression model to assess change from intake to follow-up. Inclusion criteria are summarized in the Additional file 1: Fig. 1A.

Sociodemographic characteristics of the 3418 individuals attending Safe Point between 2019 and 2021 are summarized in Table 1. Among the 3020 new clients included in the regression model evaluating risk factors at intake, 1163 (39%) reported a positive result for HCV; 55 (2%) reported a positive result for HIV; 1220 (40%) reported a previous overdose; 1305 (43%) reported accessing substance use treatment within the past year; 1191 (39%) reported mixing drugs; 1114 (37%) reported sharing injection equipment; and 923 (31%) reported using alone. Characteristics of all Safe Point participants, including those excluded from LCA and logistic regression analyses, are included in the Additional file 1: Table 1A.

**Table 1:** Sociodemographic characteristics of Safe Point participants, 2019–21. ^a^

Indicator	Aggregate sample (*n* = 3418)
Median age [IQR]	36 [30–43]
Gender, man	61% (*n* = 2091)
Ethnicity, Latinx/Hispanic	4% (*n* = 121)
Race, white	86% (*n* = 2950)
Sexual orientation, heterosexual	88% (*n* = 3000)
Housing status, homeless	36% (*n* = 1244)
Opioid use	86% (*n* = 2950)
Methamphetamine use	49% (*n* = 1660)
Cocaine use	28% (*n* = 958)
Use short needle	66% (*n* = 2239)
Using large syringe	22% (*n* = 751)
Resources for treatment/support if they want to stop using	88% (*n* = 2992)
Intention to reduce how much they use	72% (*n* = 2459)
Use interferes with their life	65% (*n* = 2236)

^a^All indicators were dichotomized to use the poLCA package in R programming [41]

Among the 377 new clients with ≥ 1 follow-up interview between 2019–2021, 196 (52%) reported testing positive for HCV; 9 (2%) reported testing positive for HIV; 134 (36%) reported an overdose at follow-up; 181 (48%) reported accessing substance use treatment at follow-up; 151 (40%) reported mixing drugs; 119 (32%) reported sharing injection equipment; and 139 (37%) reported using alone at follow-up.

### Identification of distinct client subgroups

**Fig. 1:**
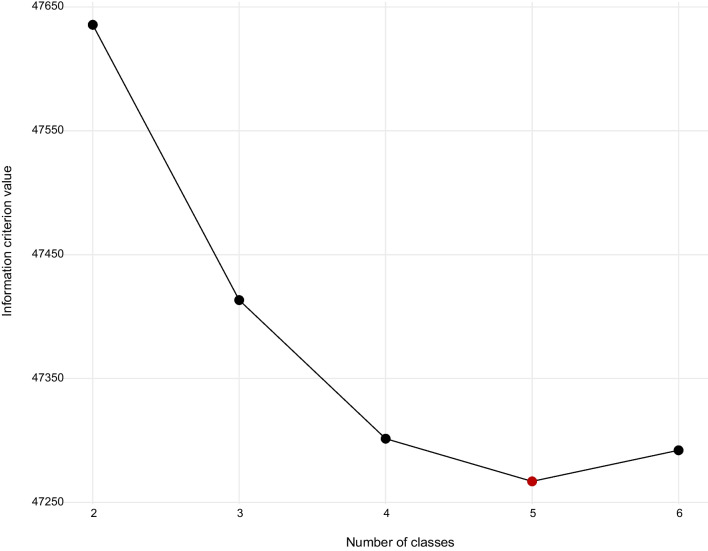
Assessment of model fit using Bayesian information criterion (BIC) values. Five models were developed, each with a different number of classes. Smaller BIC values indicate better fit. BIC value declined as the number of classes specified increased, reaching its minimum value (BIC = 47,267) with 5 classes, and then increasing by over 25 points with 6 classes. Methodologists posit that BIC differences > 10 provide strong evidence that the model with the smaller BIC value fits better [22, 53]. The red point at *x* = 5 indicates the minimum BIC value

The five-class model was selected as the final model after evaluating model fitness with BIC values (Fig. 1). Given the limited diversity of the analytic sample, a sensitivity analysis was performed, where sociodemographic indicators were not used to define latent classes. Results from the sensitivity analysis (Additional file 1: Tables 2A, 3A and 4A) also support a five-class model. Class 2 constitutes the largest share of clientele (33%) and is composed of mostly white, heterosexual men younger than 45 years of age using opioids with small syringes and a mix of short and long needles. The majority report that use is interfering with their life, they are reducing use, have resources for treatment or support, and have stable housing. This served as the reference group for logistic regression models.

Class 4 constitutes roughly one-fifth of the Safe Point population (21%) and is composed of mostly white heterosexual men and women younger than 45 years of age using opioids and methamphetamine with small syringes and short needles. The majority report that use is interfering with their life, they are reducing use, have resources for treatment or support, but half are homeless.

Class 5 makes up just under one-fifth of the clientele (18%) and is composed of mostly young white women with some diversity in sexual orientation. These participants use opioids and cocaine with small syringes and short needles and report that use is interfering with their life, they are reducing use, and they have resources for treatment or support, but a salient contingent is homeless.

Class 1 constitutes the second smallest population share (16%) and is composed of mostly homeless heterosexual men with some diversity in age and race/ethnicity. These participants use opioids and some stimulants (methamphetamine and/or cocaine), with the majority reporting that use interferes with their life and they are reducing their use, but they have few resources for treatment or support.

**Fig. 2:**
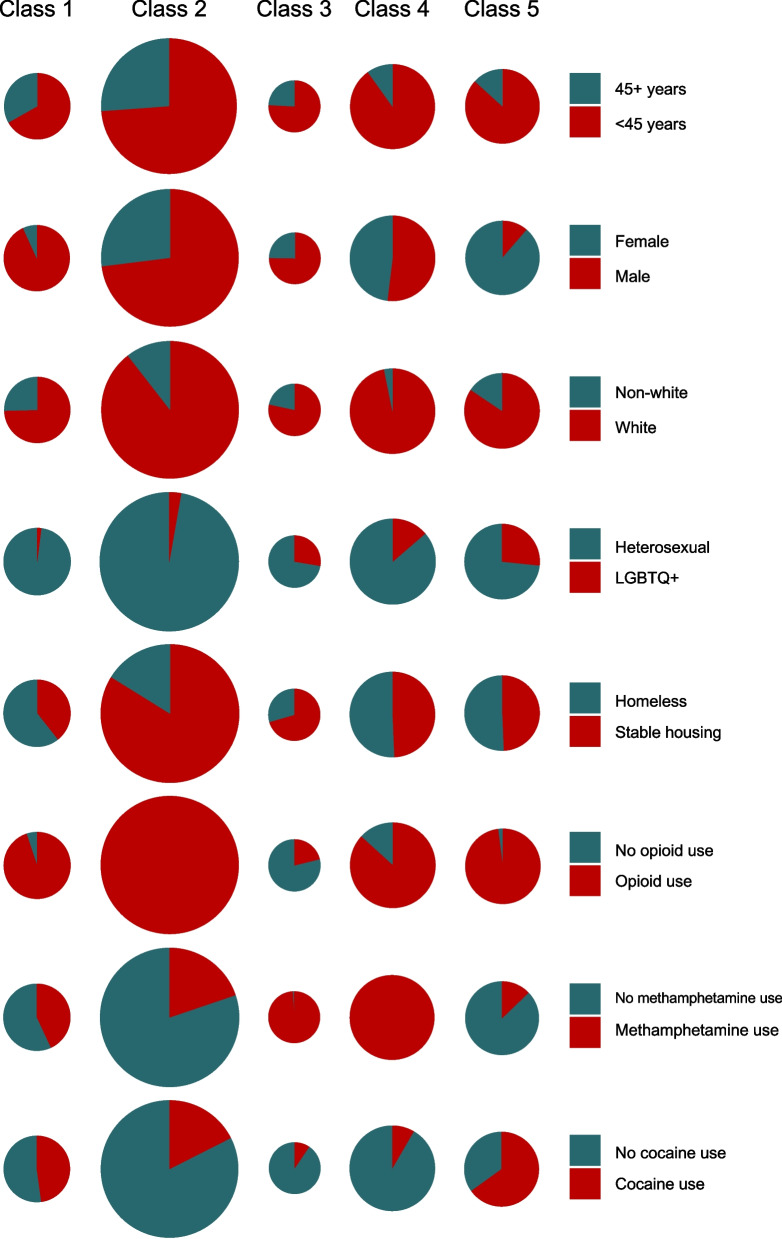
Predicted probabilities of sociodemographic characteristics, conditional on latent class membership. A five-class latent class model was developed, with salient differences in composition by age, race/ethnicity, gender, sexual orientation, housing status, and substance use. The size of graph corresponds to each class’s share of the Safe Point clientele population, with class 2 being the largest (33%), followed by class 4 (21%), class 5 (18%), class 1 (16%), and class 3 (12%). Class 1 consists of heterosexual males with housing instability and limited resources for treatment and/or support that use opioids in combination with stimulants (methamphetamine and/or cocaine). Class 2 consists of heterosexual individuals with stable housing and resources for treatment and/or support that use opioids. Class 3 consists of individuals using methamphetamine, with the most diversity in terms of race/ethnicity and sexual orientation. Class 4 consists of young (< 45 years) white individuals using opioids in combination with methamphetamine. Class 5 consists of females using opioids in combination with cocaine

Class 3 constitutes just over one-tenth of the clientele (12%) and is composed of individuals using methamphetamine with short needles and small syringes. There is some diversity in gender, sexual orientation, age, and race/ethnicity, but white, heterosexual men younger than 45 years of age predominate. The majority have stable housing and report having resources for treatment or support, with half reducing their use, but the majority do not perceive their use to interfere with their lives. The sociodemographic characteristics of each latent class are summarized in Fig. 2.

### Regression analysis: risk factors by latent class

After identifying five client subgroups, logistic regression models were developed to assess risk factors between latent classes at two distinct timepoints: (1) program intake, or enrollment and (2) follow-up (3–12 months after engaging in SSP services). Class 2 was used as the reference group, considering it constitutes the largest population share and exhibits the lowest relative risk profile, with mono-opioid use, stable housing, and resources for treatment or support.

Compared to class 2, participants in Class 4 were significantly less likely to report a history of overdose (odds ratio [OR]: 0.48 [95% confidence interval, CI: 0.36–0.64], *P* < 0.001), HCV positivity (0.56 [0.41–0.77], *P* < 0.001), and mixing drugs (0.56 [0.41–0.77], *P* < 0.001), but were more likely to report HIV positivity (16.22 [7.47–40.60], *P* < 0.001) at intake. At follow-up, their reduced likelihood of reporting positive for HCV (0.48 [0.23–0.94], *P* = 0.0359) persisted.

Participants in Class 5 were more likely, compared to class 2, to report history of substance use treatment (OR: 1.62 [95% CI: 1.30–2.01], *P* < 0.001), overdose (1.57 [1.26–1.97], *P* < 0.001), HCV positivity (1.60 [1.22–2.09], *P* < 0.001), sharing injection equipment (1.65 [1.31–2.08], *P* < 0.001), and mixing drugs (2.05 [1.63–2.58], *P* < 0.001) at intake. At follow-up, they were more likely to report accessing substance use treatment (1.80 [1.01–3.26], *P* = 0.0488) and testing positive for HCV (1.94 [1.01–3.82], *P* = 0.0497).

At intake, participants in Class 1 were more likely to report a history of substance use treatment (OR: 1.43 [95% CI: 1.18–1.73], *P* < 0.001), overdose (2.15 [1.77–2.62], *P* < 0.001), HCV positivity (1.99 [1.57–2.54], *P* < 0.001), sharing injection equipment (2.63 [2.16–3.22], *P* < 0.001), and mixing drugs (4.86 [3.96–5.97], *P* < 0.001), but less likely to report using alone (0.75 [0.61–0.92], *P* = 0.0213), compared to class 2. At follow-up, elevated risk of sharing supplies (3.65 [2.05–6.60], *P* < 0.001) and mixing drugs (2.79 [1.60–4.94], *P* < 0.001) persisted.

**Fig. 3:**
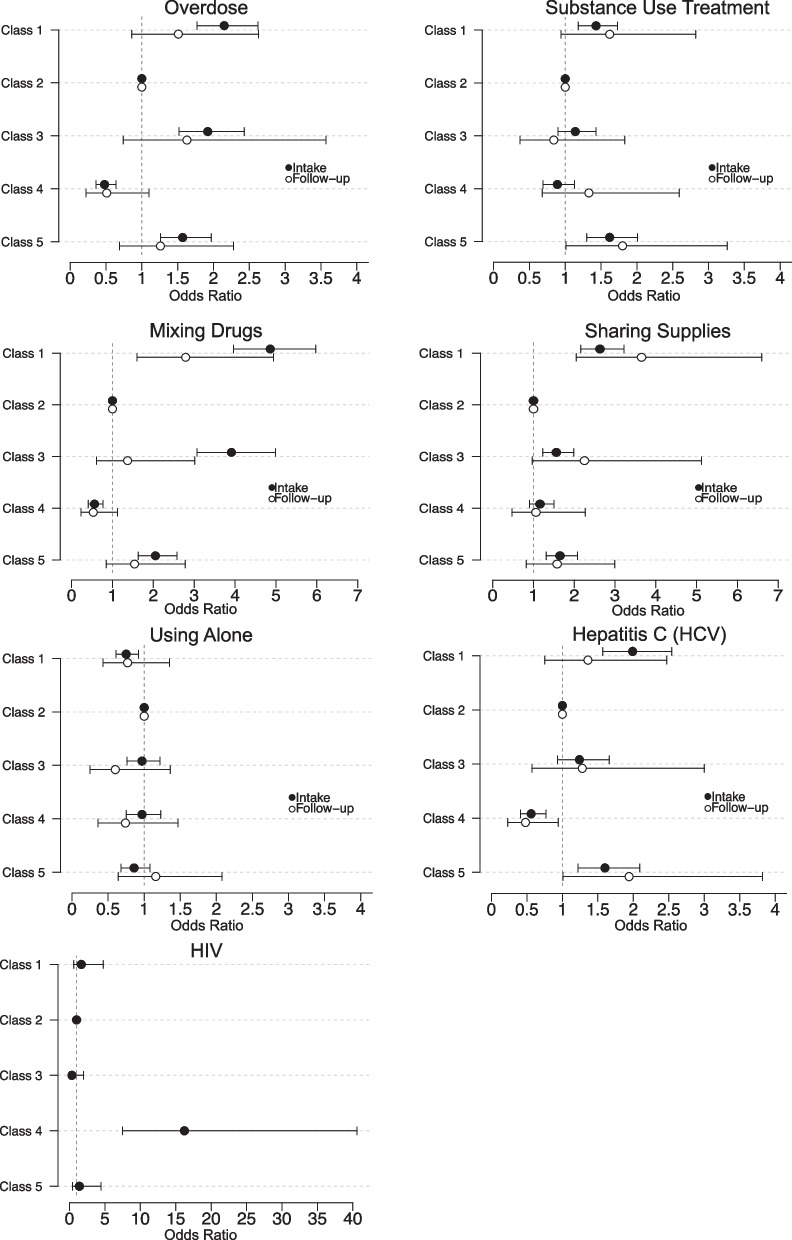
Risk factors between latent classes at intake and follow-up. At intake, differential risks of overdose, substance use treatment, mixing drugs, sharing supplies, using alone, HCV, and HIV were observed by class. Disparate risk of mixing drugs, sharing supplies, and HCV persisted at follow-up. HIV risk at follow-up was not evaluated due to limited testing results (*n* = 9)

Participants in Class 3 were more likely, compared to class 2, to report a history of overdose (OR: 1.92 [95% CI: 1.52–2.43], *P* < 0.001), sharing injection equipment (1.56 [1.23–1.99], *P* < 0.001), and mixing drugs (OR: 3.91 [95% CI: 3.07–4.99], *P* < 0.001) at intake. At follow-up, members of class 3 fared comparably, with reference to class 2, across all indicators. These differential risk factors between latent classes are summarized in Fig. 3.

## Discussion

### Summary

Five distinct typologies of participants at a SSP in Columbus, Ohio, were identified using LCA, with differential risk factors observed across latent classes at intake, some of which persisted at follow-up. Previous studies have identified differing numbers of latent classes among PWUD in their samples, but much like the sample described herein, there are three clear typologies: (1) individuals using opioids, (2) individuals using stimulants, and (3) individuals using opioids in combination with stimulants [20, 23, 31, 38]. Among Safe Point participants, the latter group could be further subdivided into individuals using opioids in combination with (a) methamphetamine and (b) cocaine.

The majority of Safe Point participants were polysubstance users, which contrasts with a recent study that found, among individuals reporting to the emergency department for overdose, mono-opioid use predominated the sample [20]. This contrast is likely due to a combination of factors, including a rapidly evolving and increasingly toxic drug supply as well as differences in toxicology screening methods and procedures across emergency departments [27, 54]. Previous LCA studies conducted in the Midwest and mid-Atlantic [38–40, 55], however, have reported a high prevalence of polydrug use, which may be due to supply-level shifts or geographic patterns of use [56]. At the time of initiating services at Safe Point, the likelihood of reporting a previous overdose and shared injection equipment was highest among individuals using opioids in combination with stimulants (Classes 1, 4, and 5), which is consistent with results from a community-based sample in Baltimore, Maryland [23]. At intake, three classes were more likely to report a recent overdose, but at follow-up, reports of overdose were comparable across latent classes, which may point to the role of SSP engagement in mitigating overdose risk among PWUD.

### Limitations

Our study has several limitations that are important to consider. We observed disparate levels of risk among SSP participants, using a dataset limited to one SSP in a large Midwestern city, so these findings may not be generalizable to rural contexts in Ohio, other Midwestern urban centers, or other regions of the USA. Despite being located in a metropolitan center, limited diversity, in terms of race, ethnicity, sexual orientation, and gender identity, was observed among Safe Point clients, which may be explained by long-standing disparities in SSP access and participation [57–59].

Additionally, the data used to assess risk at both intake and follow-up are self-reported, presenting a critical limitation. For one, the uptake of testing for HIV and HCV is rather low, even though the majority of SSPs, including Safe Point, offer on-site testing [60]. Additionally, “substance use treatment” comprises a broad array of offerings, spanning detoxification, cognitive-behavioral therapy, pharmacological interventions with medication for opioid use disorder, residential treatment, and intensive outpatient treatment [61]. Each “treatment” pathway is associated with markedly different rates of effectiveness, as medication for opioid use disorder reduces the risk of overdose [61], while the risk of overdose is heightened after release from detoxification programs due to reduced tolerance [62]. As a result, our singular measure of substance use treatment may confer protection or vulnerability to adverse outcomes (e.g., overdose).

Finally, data availability presents an additional limitation to the present study. The number of individuals excluded from analysis due to missing data was not negligible, but the characteristics of all Safe Point clients between 2019 and 2021 (Additional file 1) closely parallel the characteristics of new clients included in the analysis. This addresses concerns about representativeness of the study cohort to the SSP, but longitudinal data were only available for a fraction of SSP participant during the study period. This may be due to changes in SSP service operations, as interviews were not conducted for most of 2020, but could also reflect changes in service utilization among PWUD over the course of the COVID-19 pandemic [63]. In any case, the limited sample size limits our ability to assess how risk factors change with sustained engagement in harm reduction programming. Despite its limitations, this is the first study, to the authors’ knowledge, to make use of longitudinal data from a SSP, identifying differential risks between latent classes that persist at follow-up. Further research should be undertaken to identify structural drivers of differential risks, thereby guiding interventions that mitigate inequities.

### Potential implications for practice

This study highlights that the majority of SSP participants in Central Ohio were engaged in polysubstance use, which was associated with higher risk of overdose and infectious disease transmission, as reported previously [38]. All data on drug use were self-reported, meaning the number of individuals engaged in polysubstance use may be higher than estimates reported herein, as recent reports have noted that fentanyl use is often unintentional [64, 65], especially among people who use stimulants [66, 67]. Expanding harm reduction services, including drug checking and safe supply [68, 69], both of which have received renewed attention with the emergence of xylazine [27], may promote agency among PWUD and disrupt the toxic drug supply [69, 70], addressing the worsening overdose epidemic.

Disparate risk factors between latent classes at intake underscores that PWUD are not homogeneous and face different levels of structural vulnerability [38], which, in turn, shapes the risk environment [37]. Many of the disparities in risk were diminished at follow-up, but differential probabilities of testing positive for HCV and accessing substance use treatment persisted, calling attention to the need for interventions that overcome barriers to STI testing and treatment for SUDs, HIV, and HCV [71–73].

From a methodological standpoint, LCA provides an opportunity for harm reduction organizations to tailor their services to best meet the needs of their clients and strategize, or forecast, operational needs. By identifying unique groups within the broader population, and determining the relative size of each group, organizations can make inferences about resource (e.g., syringe types) and service needs (e.g., housing assistance, treatment referrals) to meet people where they are and address disparities in harm reduction services.

## Conclusions

Considerable heterogeneity exists among PWUD, with five distinct subgroups of clients participating in a SSP in Central Ohio. Disparate levels of risk at intake were observed between subgroups, with some risk factors persisting at follow-up. Further longitudinal investigations that yield larger sample sizes with richer diversity and geographic representation should be pursued to (1) better assess differences in outcomes among those engaged in harm reduction programming and (2) assess stability or fluidity of classes over time. LCA offers a robust methodological approach that produces useful and relevant insights to researchers and practitioners alike, providing an opportunity to identify emerging patterns and trends among PWUD and tailor harm reduction services and programming. Research and practice, in tandem, can respond to the worsening overdose epidemic, addressing its inequities and improving the lives of PWUD.

## Supplementary Information

**Additional file 1**. The supplemental file details inclusion and exclusion criteria and a sensitivity analysis removing demographic variables in the latent class analysis.

## Data Availability

The data that support the findings of this study were made available to OSU Center for HOPES, but restrictions apply to the availability of these data, which were made available through a data use agreement for the current study and are not publicly available. Data are, however, available from the authors upon reasonable request *and* with permission of Equitas Health.

## Abbreviations

AIDS: Acquired immunodeficiency syndrome
BIC: Bayesian information criterion
HCV: Hepatitis C virus
HIV: Human immunodeficiency virus
LCA: Latent class analysis
OSU: Ohio State University
PWUD: People who use drugs
SSP: Syringe service program
STI: Sexually transmitted infection
SUD: Substance use disorder
