# Supplementary material for: Differential risks of syringe service program participants in Central Ohio: a latent class analysis
Source: Harm Reduct J. 2023 Jul 28;20:97. doi: 10.1186/s12954-023-00824-8 (PMC10386257; doi:10.1186/s12954-023-00824-8)
Supplement: Supplementary file 1 — Additional file 1. The supplemental file details inclusion and exclusion criteria and a sensitivity analysis removing demographic variables in the latent class analysis. [file 12954_2023_824_MOESM1_ESM.docx]

**TITLE:** Differential risks of syringe service program participants in central Ohio: A latent class analysis

**SHORT TITLE:** Latent class analysis to characterize profiles of syringe service program clients

**AUTHORS:** Kyle J. Moon, B.S.^1^; Ian Bryant, M.A.,^1^; Anne Trinh, M.P.H.^1^; Kathryn A. Hasenstab, B.S. B.M.E.^1^; Brittany Carter, B.S.P.H.^2^; Rick Barclay^2^; Saira Nawaz, Ph.D., M.P.H.^1,3^

**AUTHOR AFFILIATIONS:**

1. Center for Health Outcomes and Policy Evaluation Studies (HOPES), The Ohio State University College of Public Health, Columbus, OH, USA
2. Equitas Health, Columbus, OH, USA
3. Division of Health Services Management and Policy, The Ohio State University College of Public Health, Columbus, OH, USA

**CORRESPONDING AUTHOR:**

Saira Nawaz, PhD, MPH

Center for Health Outcomes and Policy Evaluation Studies

Ohio State University College of Public Health

381 Cunz Hall, 1841 Neil Avenue

Columbus, OH 43210

(p) 614.292.4691 | (e) [Nawaz.16@osu.edu](mailto:Nawaz.16@osu.edu)

**SUPPLEMENTAL MATERIALS**

**Figure 1A. CONSORT flow diagram**. The original dataset provided by Safe Point included 7,890 unique individuals, 3,418 of whom were included in the LCA (4,472 were excluded due to no interview data or missing data for LCA indicators). After performing the LCA, 3,020 unique individuals were included in the first regression model to assess risk factors, as 398 were not new clients. 377 individuals, who had ≥1 interview during 2019–2021, were included in the second regression model to assess risk at follow-up.

**Table 1A. Sociodemographic characteristics of Safe Point clients, 2019–2021**. ^a^

|  | All Clients (n=7,890) | |
| --- | --- | --- |
|  | n | % |
| Gender Identity | | |
| Man | 4636 | 61.5% |
| Woman | 2904 | 38.5% |
| Age | | |
| 18–24 years | 488 | 6.3% |
| 25-34 years | 2988 | 38.7% |
| 35-44 years | 2657 | 34.4% |
| 45–54 years | 1106 | 14.3% |
| 55–64 years | 412 | 5.3% |
| 65+ years | 78 | 1.0% |
| Sexual Orientation |  |  |
| Heterosexual | 6703 | 88.6% |
| LGBTQ+ | 859 | 11.4% |
| Ethnicity |  |  |
| Hispanic/Latinx | 246 | 3.5% |
| Non-Hispanic/Latinx | 6736 | 96.5% |
| Race |  |  |
| American Indian/Alaska Native | 63 | 0.8% |
| Black | 544 | 7.1% |
| White | 6872 | 89.3% |
| Other Multiracial | 215 | 2.8% |
| Drug Use Reported |  |  |
| Opioids | 5750 | 86.2% |
| Methamphetamine | 2970 | 44.5% |
| Cocaine | 1636 | 24.5% |
| Housing Status |  |  |
| Homeless | 1064 | 30.7% |
| Stable | 2407 | 69.3% |
| Needle Length |  |  |
| Long | 1159 | 14.8% |
| Short | 2781 | 35.4% |
| Both | 3915 | 49.8% |
| Syringe Gauge Size |  |  |
| Large | 441 | 5.6% |
| Small | 6256 | 79.6% |
| Both | 1158 | 14.7% |

^a^ Percentages reflect ‘valid percentages,’ meaning missing or unknown responses are excluded from the denominator.

**Table 2A. Fit Statistics for LCA Sensitivity Analysis**. ^a^

| Number of Latent Classes | BIC |
| --- | --- |
| 2 classes | 36,772.88 |
| 3 classes | 36,664.59 |
| 4 classes | 36,623.64 |
| 5 classes | 36,600.01 |
| 6 classes | 36,623.36 |

^a^ For the sensitivity analysis, sociodemographic indicators (age, gender identity, race, and sexual orientation) were not used to define latent classes. The following indicators were used: reported substance use (opioids, cocaine, methamphetamine), housing status, needle length (short or long), syringe gauge size (small or large), use interferes with life, resources for treatment or support, trying to cut down on use, and housing status.

**Table 3A. Descriptive Characteristics of Latent Classes Derived in the Sensitivity Analysis (n=3,661)**.

|  | Class 1 | Class 2 | Class 3 | Class 4 | Class 5 |
| --- | --- | --- | --- | --- | --- |
| Opioid Use | 26.6% | 95.4% | 99.9% | 100.0% | 100.0% |
| Cocaine Use | 10.8% | 100.0% | 7.2% | 28.4% | 21.0% |
| Methamphetamine Use | 98.9% | 7.6% | 27.0% | 36.9% | 73.7% |
| Homeless | 34.0% | 39.4% | 12.5% | 49.9% | 65.0% |
| Short Needle | 78.3% | 68.5% | 59.7% | 0.0% | 89.7% |
| Large Syringe Gauge | 14.2% | 25.6% | 14.9% | 61.2% | 17.1% |
| Actively Reducing Use | 62.0% | 76.0% | 76.7% | 66.4% | 72.8% |
| Using Interferes with Life | 43.8% | 76.0% | 58.8% | 71.7% | 80.6% |
| Has Resources for Treatment/Support | 87.0% | 88.6% | 92.6% | 81.1% | 81.8% |
| **Population Share** | **17.9%** | **15.9%** | **32.4%** | **10.2%** | **23.6%** |

**Table 4A. Risk Factors at Intake, by Latent Class**. ^a,b^

|  | Class 1 | Class 2 | Class 3 | Class 4 | Class 5 |
| --- | --- | --- | --- | --- | --- |
| SUD Treatment | 0.82  [0.65 – 1.03] | 1.30  [1.06 – 1.60] * | 1.00 (ref) | 1.22  [0.93 – 1.59] | 1.34  [1.11 – 1.61] ** |
| Overdose | 0.56  [0.43 – 0.72] *** | 1.56  [1.26 – 1.92] *** | 1.00 (ref) | 1.58  [1.20 – 2.08] ** | 2.87  [2.37 – 3.48] *** |
| HCV | 0.63  [0.47 – 0.84] ** | 1.53  [1.19 – 1.97] *** | 1.00 (ref) | 1.62  [1.17 – 2.25] ** | 1.93  [1.53 – 2.43] *** |
| HIV | 14.47  [6.49 – 38.53] *** | 0.95  [0.24 – 3.37] | 1.00 (ref) | 1.58  [0.33 – 6.05] | 2.04  [0.77 – 5.95] |
| Sharing Supplies | 1.27  [1.00 – 1.61] * | 1.41  [1.13 – 1.76] ** | 1.00 (ref) | 1.68  [1.27 – 2.22] *** | 2.71  [2.23 – 3.29] *** |
| Mixing Drugs | 0.58  [0.43 – 0.76] *** | 2.78  [2.24 – 3.44] *** | 1.00 (ref) | 2.36  [1.78 – 3.11] *** | 6.33  [5.17 – 7.76] *** |
| Using Alone | 0.96  [0.76 – 1.20] | 0.98  [0.80 – 1.21] | 1.00 (ref) | 1.03  [0.78 – 1.35] | 0.83  [0.68 – 1.01] |

^a^ Data are presented as Odds Ratio [95% CI]. Odds ratios were generated via logistic regression.

^b^ * denotes P<0.05, ** denotes P<0.01, and *** denotes P<0.001. Statistical significance was assessed with the Wald test.
